# Prognostic significance of specific anti-WT1 IgG antibody level in plasma in patients with ovarian carcinoma

**DOI:** 10.1002/cam4.244

**Published:** 2014-04-08

**Authors:** Charlotta Andersson, Yusuke Oji, Nina Ohlson, Sihan Wang, Xingru Li, Ulrika Ottander, Eva Lundin, Haruo Sugiyama, Aihong Li

**Affiliations:** 1 Clinical Chemistry, Department of Medical Biosciences, Umeå UniversityUmeå, Sweden; 2 Pathology, Department of Medical Biosciences, Umeå UniversityUmeå, Sweden; 3 Department of Cancer Stem Cell Biology, Osaka University Graduate School of MedicineOsaka, Japan; 4 Department of Clinical Sciences, Obstetrics and Gynecology, Umeå UniversityUmeå, Sweden; 5 Department of Functional Diagnostic Science, Osaka University Graduate School of MedicineOsaka, Japan

**Keywords:** Ovarian carcinoma, prognosis, WT1, WT1 IgG antibody

## Abstract

Ovarian carcinoma (OC) has a poor prognosis and lack early effective screening markers. Wilm's tumor gene 1 (*WT1*) is overexpressed in OCs. Therefore, it is of great interest to investigate whether WT1-specific antibody (Ab) measurements in plasma can serve as a biomarker of anti-OC response, and is of importance in relation to patient prognosis. Peripheral blood samples were obtained from a total of 103 women with ovarian tumors with median being 1 day (range 0–48 days) before operation. WT1 IgG Ab levels were evaluated using enzyme-linked immunosorbent assay (ELISA). Immunohistochemical analysis of WT1 protein expression was performed on OC tissue samples. We found that low-WT1 Ab level in plasma was related to improved survival in patients diagnosed at stages III–IV and grade 3 carcinomas. Positive WT1 protein staining on OC tissue samples had a negative impact on survival in the entire cohort, both overall survival (OS) (*P* = 0.046) and progression-free survival (PFS) (*P* = 0.006), but not in the serous OC subtype. Combining WT1 IgG Ab levels and WT1 staining, patients with high-WT1 IgG Ab levels in plasma and positive WT1 protein staining in cancer tissues had shorter survival, with a significant association in PFS (*P* = 0.016). These results indicated that WT1 Ab measurements in plasma and WT1 staining in tissue specimens could be useful as biomarkers for patient outcome in the high-risk subtypes of OCs for postoperative individualized therapy.

## Introduction

Ovarian carcinoma (OC) is a heterogeneous disease and can be classified according to histology in major subtypes (serous, clear cell, endometrioid, mucinous and mixed epithelial, and undifferentiated). Distinct mRNA expression profiles have been identified in these subtypes 1,2. Correlation between different subtypes and chemoresistance has been reported 3,4. OC is no longer considered a single entity but as different disease processes with each subtype having distinct genetic risk factors, underlying molecular events during oncogenesis, stages at diagnosis, and responses to chemotherapy (reviewed in 5). The incidence and mortality rates of OC have slightly declined in the Nordic countries from the mid-1980s 6. However, the prognosis is still poor with 5-year relative survival around 40% in Sweden 6. In women treated for advanced (International Federation of Gynecologists and Obstetricians [FIGO]—stages III–IV) epithelial OC, the 5-year overall survival (OS) rate was only 16% compared to early stages of the disease (75%) 7,8. Age, tumor grade, FIGO clinical stage, and the amount of residual cancer after surgery are known to be prognostic factors for OC. Despite ongoing efforts to develop an effective screening strategy, only 20% of OCs are diagnosed at an early stage (stage I). The majority of patients are diagnosed at an advanced stage due to diffuse early symptoms. Transvaginal sonography (TVS), serum markers, and a combination of these two modalities have been evaluated for their ability to detect OC at early stages 9. Among the serum markers, the cancer antigen 125 (CA125) has received most attention but lacks sensitivity or specificity to be used alone 10. Therefore, effective methods for early detection are needed.

The Wilms' tumor gene 1 (*WT1*) was originally discovered as a tumor suppressor gene in Wilms' tumor, a childhood kidney neoplasm 11. We have recently demonstrated that *WT1* can act as a tumor suppressor in clear cell renal cell carcinoma (ccRCC) regulating *hTERT* gene expression via multiple pathways 12. However, subsequent research has revealed that WT1 is also involved in a number of other tumors for which WT1 might serve an oncogenic role 13. Overexpression of WT1 has been demonstrated in various human cancers including acute leukemia, breast cancer, brain tumors, and other tumors 14–18. Ovarian serous carcinoma is a known WT1 protein immunohistochemical (IHC) staining positive tumor where WT1 is used for histopathological classification 19–23 but as a prognostic factor WT1 expression may be of limited value 24–26.

Overexpressed oncogenic proteins can be considered as potential candidate antigens for cancer vaccines and T-cell therapy 27,28. WT1 seems to be an oncogenic protein involved in transcriptional regulation in leukemogenesis and for the viability of leukemia blasts 29–31. Previous studies have demonstrated that WT1 appears to be immunogenic in mice and human 32. Spontaneous development of both specific antibodies and T cells was found in patients with WT1 overexpressing tumors, suggesting that WT1 is a promising target for immunotherapeutic treatment 33–35.

Studies have demonstrated that IgM and/or IgG antibodies against WT1 were detectable at a higher level in sera in patients with leukemia and myelodysplastic syndrome (MDS) compared with healthy individuals 33,36. Moreover, high levels of anti-WT1 antibody (Ab) in serum were found to be a prognostic factor of longer survival in patients with MDS and in patients with non-small cell lung cancer (NSCLC) 37,38.

In this study, our main goal was to investigate the importance of WT1-specific IgG Ab in plasma as a marker of anti-OC immune response and possible relation to disease progression. Furthermore, we wanted to determine whether WT1 IgG Ab level in plasma may relate to WT1 protein expression in cancer tissues specimens.

## Material and Methods

### Patients and material

The study included a total of 103 ovarian specimens from patients undergoing surgery at the Department of Obstetrics and Gynaecology, Umeå University Hospital, Sweden, between August 1993 and November 2000. All tissue specimens and peripheral blood were collected under a protocol approved by the Human Ethics Committee, Umeå University (Dnr 06-057M). Informed consent was obtained from all patients. Plasma samples were obtained from patients with median being 1 day (range 0–48 days) before operation and stored at −80°C until use. Formalin-fixed, paraffin-embedded tissue sections were used for IHC detection of WT1. The histological grading was determined by pathologists according to the WHO classification. The FIGO stages were classified according to staging system (http://www.figo.org/publications/cancer_staging_classification). Medical records of the patients during follow up and the Swedish Cause of Death Register were retrospectively reviewed and used for identification of progression-free survival (PFS) and OS analysis. Patients in this study did not receive any radiation and/or chemotherapy before operation. Characteristics of patients with ovarian tumors are presented in Table1.

**Table 1:** Characteristics of patients with ovarian tumors

Patient characteristics	Number of patients (%)
Age (years)	
Median	56
Range	20–81
Histological classification	
Ovarian carcinoma (OC)	52 (50.5%)
Serous adenocarcinoma	33 (63.5%)
Endometrioid adenocarcinoma	8 (15.4%)
Mucinous adenocarcinoma	5 (9.6%)
Clear cell carcinoma	2 (3.8%)
Mixed epithelial tumors	1 (1.9%)
Undifferentiated carcinoma	3 (5.8%)
Borderline tumors	18 (17.5%)
Benign tumors	33 (32%)
FIGO stage	
I	14 (13.6%)
II	4 (3.9%)
III	24 (23.3%)
IV	10 (9.7%)
Tumor grade	
1	7 (6.8%)
2	16 (15.5%)
3	26 (25.2%)
Follow up (months)	
Median	90.5
Range	0.9–228.8

FIGO, International Federation of Gynecology and Obstetrics.

### Immunohistochemistry

WT1 staining by IHC is known to be used for differential diagnosis in malignant ovarian tumors. Usage of WT1 staining in the diagnostic setting for benign ovarian tumors has not been reported due to negative WT1 staining in most of benign ovarian tumors. Regarding WT1 expression in borderline tumors, about 10–16% of borderline ovarian tumors were reported with positive WT1 staining 39,40. In this study, WT1 protein expression using IHC was analyzed on only malignant tumor specimens. Formalin-fixed and paraffin-embedded tissue specimens were cut (4-*μ*m thick sections) and mounted on glass slides. Sections were stained with monoclonal WT1 Ab (clone 6F-H2, Dako, Carpinteria, CA) in a dilution of 1:50 using a fully automated slide preparation system (Ventana Benchmark XT; Ventana Medical Systems, Inc., Tucson, AZ). The intensity of WT1 expression was classified as nonstaining, weak and intensive as previously described 22. Tumors with heterogeneous intensity of WT1 were classified according to the highest degree of immunoreactivity if it occupied more than 10% of the tumor.

### Enzyme-linked immunosorbent assay

WT1 IgG Ab titers were measured by the method described previously 37 with minor modifications. In brief, enzyme-linked immunosorbent assay (ELISA) 96-well plates were coated with 100 *μ*L of three recombinant glutathione S-transferase tagged, WT1 fragment proteins, WT-Fr1 (1–182 aa), WT-Fr2 (180–324 aa), and WT-Fr3 (318–449 aa) (0.6 *μ*g/well for each WT1 fragment protein) in immobilization buffer (10 mmol/L NaCO_3_, 30 mmol/L NaHCO_3_, 0.02% NaN_3_, pH 9.6) overnight at 37°C. Then, plates were washed with tris-buffered saline (TBS) and blocked with blocking solution (TBS containing 0.05% Tween 20 and 1% gelatin) at 37°C for 2 h. Plasma were diluted at 1:100 in blocking solution. Thus, 100 *μ*L of blocking solution was used as the negative control for the assay. Then, 100 *μ*L of the diluted plasma (1:50 dilution) was added to each well for overnight incubation at 4°C. Plates were washed with TBS containing 0.05% Tween-20 and incubated with ALP-conjugated goat anti-human IgG Ab (Santa Cruz Biotechnology, Dallas, TX) diluted at 1:500 in TBS containing 1% Tween-20 for 2 h at room temperature. After washing, bound WT1 IgG Ab was visualized for each well using 100 *μ*L of BCIP-NBT kit (Nacalai Tesque, Kyoto, Japan). Then, absorbance at 550 nm was measured using a microplate reader MTP-310 (Corona Electric, Ibaraki, Japan). The absorbance for sample plasma was calculated by subtracting absorbance of the negative control from measured absorbance of the sample. All samples were examined in duplicate.

The titers of WT1 IgG Ab were calculated by interpolation from the corresponding standard line which was constructed for each assay from the results of simultaneous measurements of serial dilutions of anti-WT1 C19 Ab (8, 40, 200, and 1000 ng/mL), using ALP-conjugated goat anti-rabbit IgG Ab (diluted at 1:500; Santa Cruz Biotechnology) as the second Ab. WT1 Ab titers in the plasma that produced the absorbance at 550 nm equal to that produced by 0.1 *μ*g/mL of anti-WT1 C19 Ab was defined as a 1.0 WT1-reacting-unit (WRU) in the ELISA system.

When samples demonstrated high titers of WT1 IgG Ab showing out of measurable range in the ELISA system, these samples were diluted with blocking solution to measurable levels and reanalyzed.

### Statistical analysis

Statistical analysis was performed using SPSS PASW Statistics statistical software, version 18 (SPSS, Chicago, IL). The Mann–Whitney *U* test was used to compare differences between two independent variables. Correlations between two variables were tested according to Spearman correlation test. Any *P*-value of less than 0.05 was taken to represent a statistically significant difference. The Kaplan–Meier method was used to estimate the distribution of PFS and OS. The log-rank test was used to determine differences in survival between groups.

## Results

### WT1-specific IgG Ab in plasma in patients with ovarian tumors

**Figure 1:**
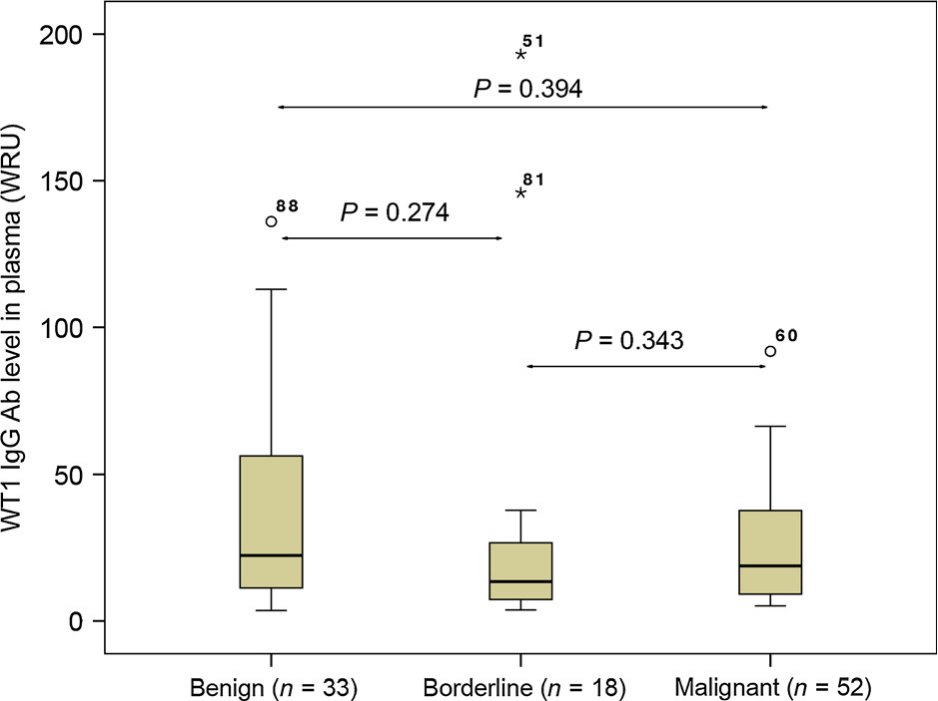
Box plots show levels of WT1 IgG Ab titers in plasma samples in different groups of patients with ovarian tumors. No differences in WT1 IgG Ab titers are detected in plasma samples between patients with malignant, borderline, and benign ovarian tumors.

WT1 IgG Ab titers in plasma were evaluated using ELISA in a total of 103 women with ovarian tumors (52 malignant OCs, 18 borderline tumors, 33 benign tumors). WT1 IgG Ab was detected in all plasma samples, with a range from 3.6 to 1841.6 (median 18.2). No differences in WT1 Ab level were found between malignant, borderline, and benign tumors (Fig.1). Using cut-off at the median (18.8) of WT1 IgG Ab levels obtained from 52 OC samples, patients were divided into subgroups with high level (≥median) and low level (<median). No significant difference was found between WT1 IgG Ab levels and clinical parameters including age, histological subtype, FIGO stage, grade, disease progression, and OS (Table2).

**Table 2:** WT1 IgG level and WT1 protein expression in ovarian carcinoma

	WT1 Ab in plasma by ELISA				WT1 protein by IHC			
*n*	WT1 <18.8 WRU	WT1 ≥18.8 WRU	*P*	*n*	Negative	Positive	*P*
Age	52			1	50			0.476
<50 years	12	6 (11.5%)	6 (11.5%)		11	4 (8.0%)	7 (14.0%)	
≥50 years	40	20 (38.5%)	20 (38.5%)		39	10 (20.0%)	29 (58.0%)	
Histological subtype				0.840				<0.001
Serous	33	16 (30.8%)	17 (32.7%)		31	1 (2%)	30 (60%)	
Mucinous	5	3 (5.8%)	2 (3.8%)		5	5 (10.0%)	0	
Endometrioid	8	3 (5.8%)	5 (9.6%)		8	4 (8.0%)	4 (8.0%)	
Clear cell	2	1 (1.9%)	1 (1.9%)		2	2 (4.0%)	0	
Mixed epithelial	1	1 (1.9%)	0		1	1 (2.0%)	0	
Undifferentiated/unclassified	3	2 (3.8%)	1 (1.9%)		3	1 (2.0%)	2 (4.0%)	
FIGO stage				1				<0.001
Early (I–II)	18	9 (17.3%)	9 (17.3%)		17	11 (22.0%)	6 (12.0%)	
Advanced (III–IV)	34	17 (32.7%)	17 (32.7%)		33	3 (6.0%)	30 (60.0%)	
Tumor grade				0.94				0.007
1	7	3 (6.1%)	4 (8.2%)		7	3 (6.4%)	4 (8.5%)	
2	16	8 (16.3%)	8 (16.3%)		14	7 (14.9%)	7 (14.9%)	
3	26	13 (26.5%)	13 (26.5%)		26	2 (4.3%)	24 (51.1%)	
Disease progression				0.27				0.005
Event	27	11 (21.2%)	16 (30.8%)		27	3 (6.0%)	24 (48.0%)	
Event free	25	15 (28.8%)	10 (19.2%)		23	11 (22.0%)	12 (24.0%)	
Overall survival				0.237				0.016
Survive	17	11 (21.2%)	6 (11.5%)		15	8 (16.0%)	7 (14.0%)	
Died	35	15 (28.8%)	20 (38.5%)		35	6 (12.0%)	29 (58.0%)	

Ab, antibody; ELISA, enzyme-linked immunosorbent assay; IHC, immunohistochemistry; FIGO, International Federation of Gynecology and Obstetrics.

### A low level of WT1 IgG Ab is associated with favorable prognosis in patients diagnosed at advanced stage and high-grade carcinomas

**Figure 2:**
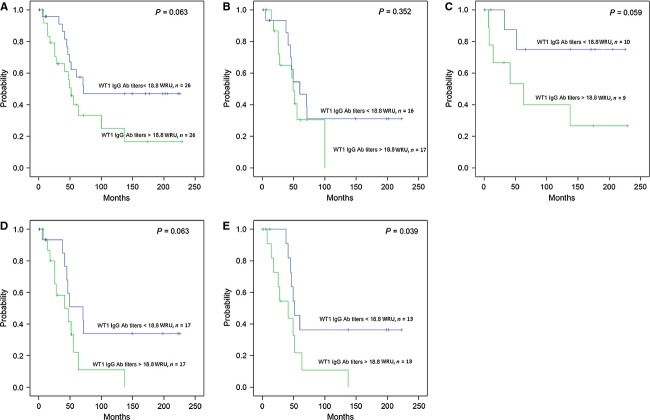
Kaplan–Meier plot of the progression-free survival (PFS) in patients with ovarian carcinoma. Patients are grouped based on Wilms' tumor gene 1 (WT1) IgG Ab titer level. Median (18.8 WRU) is used for a cut-off level. (A) PFS for entire cohort of ovarian carcinoma patients. (B) PFS for patients with serous ovarian carcinoma. (C) PFS for patients with nonserous subtypes. (D) PFS for patients diagnosed with advanced clinical stages III or IV. (E) PFS for patients identified with grade 3.

To determine the prognostic relevance of WT1 IgG Ab level detected in plasma before operation, the patients with OCs were divided into two groups according to the median as described above. WT1 IgG Ab level was not shown to be of prognostic significance in OS (*P* = 0.129, data not shown in figure) but a trend that low level of WT1 Ab was correlated with a longer PFS (*P* = 0.063, Fig.2A). There was no significant difference in OS (*P* = 0.273, data not shown in Figure) or PFS (*P* = 0.352, Fig.2B) with regard to WT1 IgG Ab level in serous OC whereas patients with nonserous subtypes showed a trend to a poor clinical outcome correlated with high-WT1 IgG Ab level for PFS (*P* = 0.059, Fig.2C). The 18 OC patients diagnosed in early stages (I–II) had no significant differences in OS and PFS regardless of WT1 IgG Ab level (*P* = 0.124 and *P* = 0.432, data not shown in figure). No significant difference in OS (*P* = 0.318) but a trend toward favorable PFS was observed in patients with OCs at advanced stages (III–IV) and low-WT1 IgG Ab levels (*P* = 0.063, Fig.2D). Similarly, 23 patients with grades 1 and 2 did not differ in outcome in relation to WT1 IgG Ab levels (*P* = 0.832, data not shown in Figure). However, in the 26 women with a grade 3 OC, low-WT1 IgG Ab levels were related to a slightly improved OS (*P* = 0.053) and significantly better PFS (*P* = 0.039, Fig.2E) compared with high-WT1 IgG Ab levels. The results indicated that in advanced stages (III–IV) or grade 3 OCs, a low level of WT1 IgG Ab was related with a favorable prognosis.

### WT1 protein expression in tumor specimens in patients with OCs

**Figure 3:**
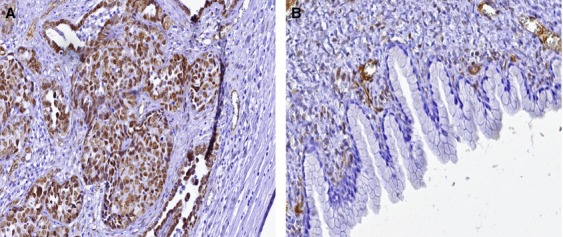
Immunohistochemical detection of the WT1 protein showed nuclear immunoreactivity in epithelial cells in ovarian serous adenocarcinoma (A) and negative staining in benign ovarian mucinous cystadenoma (B). Original magnification 400×.

IHC analysis of WT1 protein expression was performed on tissue specimens from 50 of 52 OC because two tissue samples were not available. WT1 was dominantly stained in nuclei of ovarian cancer cells. Strong WT1 protein staining was demonstrated in 35 of 50 specimens and weak WT1 staining was observed in only one tissue sample. Figure3 showed examples for WT1-positive staining in ovarian serous adenocarcinoma and negative staining in benign mucinous cystadenoma. Correlations between WT1 protein staining in OC tissue samples and clinical pathological parameters are summarized in Table2. Positive WT1 protein staining was found more frequently in serous histological subtype (60%) than nonserous (12%, *P* < 0.001). Advance stage of the disease (FIGO stages III and IV) was related to positive WT1 staining (*P* < 0.001), as well as grade 3 compared to grades 1 and 2 (*P* = 0.007). Poor outcome was observed in patients with positive WT1 staining both for disease progression (*P* = 0.005) and OS (*P* = 0.016). No significant differences were observed between patients with positive versus negative WT1 staining with regard to age (Table2).

### Positive WT1 protein staining correlates with poor clinical outcome

**Figure 4:**
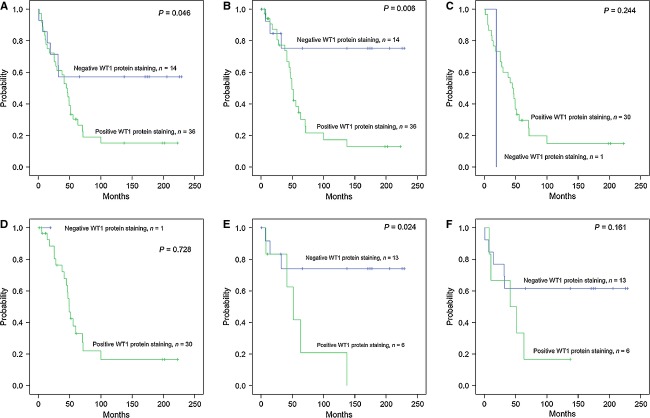
Kaplan–Meier plot of the survival in patients with ovarian carcinoma. Patients are grouped according to immunohistochemistry (IHC) staining (positive or negative) of the Wilms' tumor gene 1 (WT1). (A) Overall survival (OS) for 50 patients studied. (B) Progression-free survival (PFS) for patients included in the study. (C) OS for patients with serous ovarian carcinoma. (D) PFS for patients with serous ovarian carcinoma. (E) PFS for patients with nonserous subtypes. (F) OS for patients with nonserous subtypes.

Kaplan–Meier analysis showed that patients with positive WT1 protein staining in OCs had shorter survival for both OS (*P* = 0.046, Fig.4A) and PFS (*P* = 0.006, Fig.4B). When stratified by subtype, there was no significant difference in OS or PFS with regard to WT1 protein expression in serous OC (*P* = 0.244, Fig.4C; *P* = 0.728, Fig.4D), whereas patients with nonserous subtypes showed poor clinical outcome correlated with positive WT1 protein staining for PFS (*P* = 0.024, Fig.4E) but not OS (*P* = 0.161, Fig.4F). The results demonstrated that positive WT1 staining was associated with worse prognosis in patients with nonserous subtypes but not in those with serous subtype.

### Prognostic impact by combining WT1 IgG Ab level in plasma and WT1 protein staining in OC specimens

**Figure 5:**
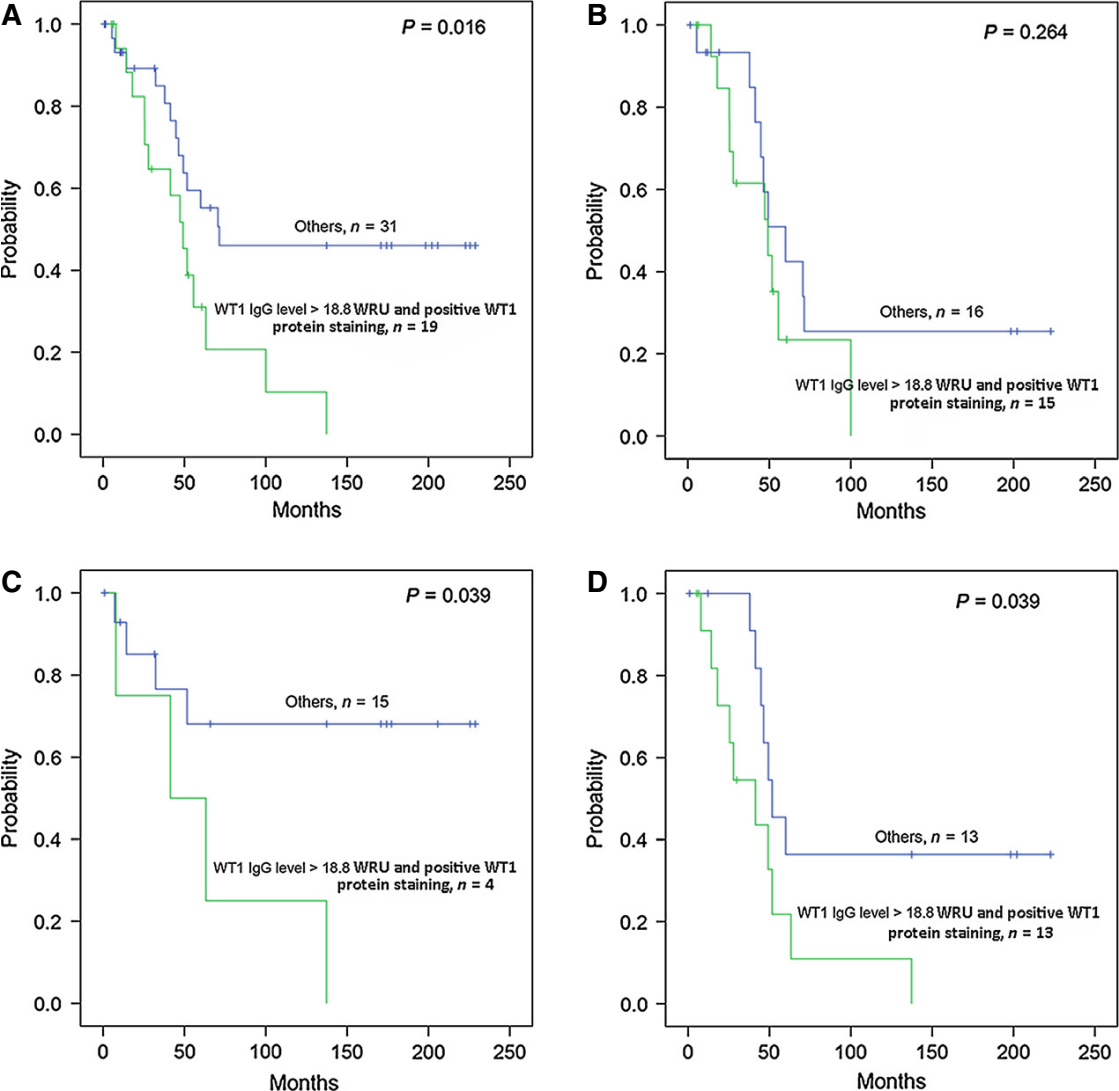
Kaplan–Meier plot of the progression-free survival (PFS) in patients with ovarian carcinoma. Patients are grouped by combing WT1 IgG Ab levels detected in plasma and WT1 protein staining in ovarian carcinoma specimens. (A) Poorer PFS for patients with high-WT1 IgG Ab level and positive WT1 protein staining compared with others including patients with low-IgG Ab level and/or WT1 staining negative. (B) PFS for serous ovarian carcinoma patients with high-WT1 IgG Ab level and positive WT1 protein staining compared with others including patients with low-IgG Ab level and/or WT1 staining negative. (C) PFS for nonserous subtypes of patients with high-WT1 IgG Ab level and positive WT1 protein staining compared with others including patients with low-IgG Ab level and/or WT1 staining negative. (D) PFS for patients diagnosed with grade 3. The log-rank test shows significant differences in PFS between with high-WT1 IgG Ab level and positive WT1 protein staining and others including patients with low IgG Ab level and/or WT1 staining negative.

In 50 OC patients data for both IHC for WT1 protein staining and IgG Ab titer in plasma were collected. Patients were divided into subgroups based on WT1 IHC staining. Median value of WT1 Ab level was 21 (range 5.4–365.7) for the 36 patients with positive WT1 protein staining and 16 (range 5.2–46.9) for the 14 women with negative staining. No correlation between WT1 IgG Ab and WT1 staining was found (*P* = 0.280). Survival analysis in the subgroup with high-WT1 IgG Ab levels showed that positive WT1 protein staining was related to shorter survival, significantly associated in PFS (*P* = 0.016, Fig.5A). In serous OC, patients with both high-WT1 IgG Ab level and WT1-positive staining survival did not differ in PFS compared with others (*P* = 0.264, Fig.5B), whereas significant poor PFS was found in subgroups with nonserous subtype (*P* = 0.039, Fig.5C) and tumor grade 3 (*P* = 0.039, Fig.5D). However, in the low-WT1 IgG Ab group, WT1 protein staining did not have any impact on outcome (data not shown).

## Discussion

In this study, we found that a low level of WT1 IgG Ab in plasma was associated with favorable prognosis in patients with grade 3 OCs. Positive WT1 protein staining by IHC in cancer tissue specimens was found more frequently in advanced stages, grade 3 and serous OCs. Positive WT1 staining was related with poorer survival in subtypes of the nonserous carcinomas, but not in the group of serous carcinomas. Furthermore, patients with high-WT1 IgG level in plasma and positive WT1 staining in cancer tissue specimens had a shorter survival than those with high-WT1 IgG Ab level but negative WT1 staining.

A previous study of WT1 IgG Ab in NSCLC showed that the level of WT1 IgG Ab was elevated in NSCLC patients, indicating a humoral immune response against cancer-derived WT1 protein 37. In NSCLC and hematopoetic malignancies, WT1-specific immune response was observed to be biased toward the Th1-type cells 37,41. Similarly to our results in OCs, the NSCLC study demonstrated no correlation between WT1 Ab levels and clinical parameters 37. However, previous studies observed high-WT1 Ab levels to be associated with longer survival in both NSCLC and MDS 37,38. Here, on the other hand, we showed in OC that low-WT1 IgG Ab levels were associated with improved clinical outcome in stages III and IV or grade 3 subgroups. The reason for this is unclear. It may reflect the difference in the immune response against WT1 protein in individual OC patients. Recently, clinical trials of WT1 peptide-based immunotherapy have been conducted in various malignancies including renal cell carcinoma, multiple myeloma, acute myeloid leukemia, lung cancer, and MDS 42–47. Suppressed tumor growth, decrease in the amount of cancer cells, and reduced level of tumor marker have been reported as effects of WT1 peptide postvaccination 42–47. Data from the clinical trials suggest that a WT1-specific immune response may be developed in patients having received the WT1-peptide immunotherapy. More interestingly, a recent study used intracellular WT1 as a target for treatment with monoclonal antibody recognizing a peptide fragment of WT1, called ESK1 48. ESK1 bound to several leukemia, solid tumor cell lines and primary leukemia cells, and mediated antibody-dependent human effector cell cytotoxicity in vitro. Low doses of ESK1 antibody cleared established, disseminated, human acute lymphocytic leukemia and Philadelphia chromosome-positive leukemia in mouse models. These findings indicate that ESK1 can be a potential therapeutic agent for a wide range of cancers overexpressing the WT1 oncoprotein. For OC patients with high expression of WT1, this antibody therapy may have a future clinical impact as a potential therapeutic alternative.

In accordance with previous reports by Hogdall et al. and Hylander et al. 24,25, we showed that WT1 expression related to advanced stages and histological grade. In contrast, a study by Netinatsunthorn et al. mainly composed of advanced stage III and IV tumors found no correlation between WT1 IHC expression and tumor stage or tumor grade 49. An association between higher WT1 IHC expression and the serous OC subtype has been demonstrated in previous studies and confirmed in this study 22,26,50,51. Poor survival was correlated with positive WT1 expression, which was observed in this study as well as previous studies 24,26. Kobel et al. showed that WT1 is a favorable prognostic marker in the high-grade serous OC 26. While we found no impact on survival within the serous subtype, the nonserous subgroup showed a correlation similar to that of the entire cohort. These findings support the hypothesis that histologic subtypes in OC differ considerably in characteristics.

Interestingly, we found no correlation between WT1 IHC staining and WT1 Ab levels. In the high-WT1 Ab subgroup, positive WT1 staining was observed to be associated with shorter survival, which was not the case in the low-WT1 Ab group. Although no collected data regarding this relationship was found in OCs, Tamura et al. observed that in MDS, WT1 Ab levels were not correlated to WT1 mRNA expression in plasma, indicating that the immune system could recognize cancer-derived WT1 protein and response to it independent on the amount of cancer cells 38.

In summary, our study has shown that low-IgG Ab levels are of positive prognostic significance in grade 3 OCs while positive WT1 expression by IHC has a negative impact on survival in the subgroup consisting of nonserous OCs with high-WT1 Ab levels. These results suggest that WT1 IgG Ab levels may be used as a prognostic marker in addition to WT1 staining in stratification/classification, particularly in the subgroup of patients with nonserous OC and grade 3. Considering the limited patient material, the prognostic impact needs to be verified in larger study populations.

## References

[b1] Schwartz DR, Kardia SL, Shedden KA, Kuick R, Michailidis G, Taylor JM (2002). Gene expression in ovarian cancer reflects both morphology and biological behavior, distinguishing clear cell from other poor-prognosis ovarian carcinomas. Cancer Res.

[b2] Zorn KK, Bonome T, Gangi L, Chandramouli GV, Awtrey CS, Gardner GJ (2005). Gene expression profiles of serous, endometrioid, and clear cell subtypes of ovarian and endometrial cancer. Clin. Cancer Res.

[b3] du Bois A, Luck HJ, Meier W, Adams HP, Mobus V, Costa S (2003). A randomized clinical trial of cisplatin/paclitaxel versus carboplatin/paclitaxel as first-line treatment of ovarian cancer. J. Natl. Cancer Inst.

[b4] Jazaeri AA, Awtrey CS, Chandramouli GV, Chuang YE, Khan J, Sotiriou C (2005). Gene expression profiles associated with response to chemotherapy in epithelial ovarian cancers. Clin. Cancer Res.

[b5] Gurung A, Hung T, Morin J, Gilks CB (2013). Molecular abnormalities in ovarian carcinoma: clinical, morphological and therapeutic correlates. Histopathology.

[b6] Klint A, Engholm G, Storm HH, Tryggvadottir L, Gislum M, Hakulinen T (2010). Trends in survival of patients diagnosed with cancer of the digestive organs in the Nordic countries 1964–2003 followed up to the end of 2006. Acta Oncol.

[b7] Skirnisdottir I, Sorbe B (2007). Prognostic factors for surgical outcome and survival in 447 women treated for advanced (FIGO-stages III-IV) epithelial ovarian carcinoma. Int. J. Oncol.

[b8] Skirnisdottir I, Sorbe B (2008). Prognostic impact of body mass index and effect of overweight and obesity on surgical and adjuvant treatment in early-stage epithelial ovarian cancer. Int. J. Gynecol. Cancer.

[b9] Das PM, Bast RC (2008). Early detection of ovarian cancer. Biomark. Med.

[b10] Bast RC (2003). Status of tumor markers in ovarian cancer screening. J. Clin. Oncol.

[b11] Haber DA, Buckler AJ, Glaser T, Call KM, Pelletier J, Sohn RL (1990). An internal deletion within an 11p13 zinc finger gene contributes to the development of Wilms' tumor. Cell.

[b12] Sitaram RT, Degerman S, Ljungberg B, Andersson E, Oji Y, Sugiyama H (2010). Wilms' tumour 1 can suppress hTERT gene expression and telomerase activity in clear cell renal cell carcinoma via multiple pathways. Br. J. Cancer.

[b13] Yang L, Han Y, Suarez Saiz F, Minden MD (2007). A tumor suppressor and oncogene: the WT1 story. Leukemia.

[b14] Inoue K, Ogawa H, Sonoda Y, Kimura T, Sakabe H, Oka Y (1997). Aberrant overexpression of the Wilms tumor gene (WT1) in human leukemia. Blood.

[b15] Loeb DM, Evron E, Patel CB, Sharma PM, Niranjan B, Buluwela L (2001). Wilms' tumor suppressor gene (WT1) is expressed in primary breast tumors despite tumor-specific promoter methylation. Cancer Res.

[b16] Menssen HD, Renkl HJ, Rodeck U, Maurer J, Notter M, Schwartz S (1995). Presence of Wilms' tumor gene (wt1) transcripts and the WT1 nuclear protein in the majority of human acute leukemias. Leukemia.

[b17] Nakatsuka S, Oji Y, Horiuchi T, Kanda T, Kitagawa M, Takeuchi T (2006). Immunohistochemical detection of WT1 protein in a variety of cancer cells. Mod. Pathol.

[b18] Silberstein GB, Van Horn K, Strickland P, Roberts CT, Daniel CW (1997). Altered expression of the WT1 Wilms tumor suppressor gene in human breast cancer. Proc. Natl. Acad. Sci. USA.

[b19] Goldstein NS, Bassi D, Uzieblo A (2001). WT1 is an integral component of an antibody panel to distinguish pancreaticobiliary and some ovarian epithelial neoplasms. Am. J. Clin. Pathol.

[b20] Madore J, Ren F, Filali-Mouhim A, Sanchez L, Kobel M, Tonin PN (2010). Characterization of the molecular differences between ovarian endometrioid carcinoma and ovarian serous carcinoma. J. Pathol.

[b21] McCluggage WG (2008). My approach to and thoughts on the typing of ovarian carcinomas. J. Clin. Pathol.

[b22] Shimizu M, Toki T, Takagi Y, Konishi I, Fujii S (2000). Immunohistochemical detection of the Wilms' tumor gene (WT1) in epithelial ovarian tumors. Int. J. Gynecol. Pathol.

[b23] Soslow RA (2008). Histologic subtypes of ovarian carcinoma: an overview. Int. J. Gynecol. Pathol.

[b24] Hogdall EV, Christensen L, Kjaer SK, Blaakaer J, Christensen IJ, Gayther S (2007). Expression level of Wilms tumor 1 (WT1) protein has limited prognostic value in epithelial ovarian cancer: from the Danish “MALOVA” ovarian cancer study. Gynecol. Oncol.

[b25] Hylander B, Repasky E, Shrikant P, Intengan M, Beck A, Driscoll D (2006). Expression of Wilms tumor gene (WT1) in epithelial ovarian cancer. Gynecol. Oncol.

[b26] Kobel M, Kalloger SE, Boyd N, McKinney S, Mehl E, Palmer C (2008). Ovarian carcinoma subtypes are different diseases: implications for biomarker studies. PLoS Med.

[b27] Gao L, Bellantuono I, Elsasser A, Marley SB, Gordon MY, Goldman JM (2000). Selective elimination of leukemic CD34(+) progenitor cells by cytotoxic T lymphocytes specific for WT1. Blood.

[b28] Ohminami H, Yasukawa M, Fujita S (2000). HLA class I-restricted lysis of leukemia cells by a CD8(+) cytotoxic T-lymphocyte clone specific for WT1 peptide. Blood.

[b29] Algar EM, Khromykh T, Smith SI, Blackburn DM, Bryson GJ, Smith PJ (1996). A WT1 antisense oligonucleotide inhibits proliferation and induces apoptosis in myeloid leukaemia cell lines. Oncogene.

[b30] Svedberg H, Richter J, Gullberg U (2001). Forced expression of the Wilms tumor 1 (WT1) gene inhibits proliferation of human hematopoietic CD34(+) progenitor cells. Leukemia.

[b31] Yamagami T, Sugiyama H, Inoue K, Ogawa H, Tatekawa T, Hirata M (1996). Growth inhibition of human leukemic cells by WT1 (Wilms tumor gene) antisense oligodeoxynucleotides: implications for the involvement of WT1 in leukemogenesis. Blood.

[b32] Gaiger A, Reese V, Disis ML, Cheever MA (2000). Immunity to WT1 in the animal model and in patients with acute myeloid leukemia. Blood.

[b33] Gaiger A, Carter L, Greinix H, Carter D, McNeill PD, Houghton RL (2001). WT1-specific serum antibodies in patients with leukemia. Clin. Cancer Res.

[b34] Scheibenbogen C, Letsch A, Thiel E, Schmittel A, Mailaender V, Baerwolf S (2002). CD8 T-cell responses to Wilms tumor gene product WT1 and proteinase 3 in patients with acute myeloid leukemia. Blood.

[b35] Vermeij R, de Bock GH, Leffers N, Ten Hoor KA, Schulze U, Hollema H (2011). Tumor-infiltrating cytotoxic T lymphocytes as independent prognostic factor in epithelial ovarian cancer with Wilms tumor protein 1 overexpression. J. Immunother.

[b36] Elisseeva OA, Oka Y, Tsuboi A, Ogata K, Wu F, Kim EH (2002). Humoral immune responses against Wilms tumor gene WT1 product in patients with hematopoietic malignancies. Blood.

[b37] Oji Y, Kitamura Y, Kamino E, Kitano A, Sawabata N, Inoue M (2009). WT1 IgG antibody for early detection of nonsmall cell lung cancer and as its prognostic factor. Int. J. Cancer.

[b38] Tamura H, Dan K, Yokose N, Iwakiri R, Ohta M, Sakamaki H (2010). Prognostic significance of WT1 mRNA and anti-WT1 antibody levels in peripheral blood in patients with myelodysplastic syndromes. Leuk. Res.

[b39] Cathro HP, Stoler MH (2005). The utility of calretinin, inhibin, and WT1 immunohistochemical staining in the differential diagnosis of ovarian tumors. Hum. Pathol.

[b40] Zhao C, Bratthauer GL, Barner R, Vang R (2007). Diagnostic utility of WT1 immunostaining in ovarian sertoli cell tumor. Am. J. Surg. Pathol.

[b41] Wu F, Oka Y, Tsuboi A, Elisseeva OA, Ogata K, Nakajima H (2005). Th1-biased humoral immune responses against Wilms tumor gene WT1 product in the patients with hematopoietic malignancies. Leukemia.

[b42] Iiyama T, Udaka K, Takeda S, Takeuchi T, Adachi YC, Ohtsuki Y (2007). WT1 (Wilms' tumor 1) peptide immunotherapy for renal cell carcinoma. Microbiol. Immunol.

[b43] Oka Y, Tsuboi A, Fujiki F, Shirakata T, Nishida S, Hosen N (2008). “Cancer antigen WT1 protein-derived peptide”-based treatment of cancer -toward the further development. Curr. Med. Chem.

[b44] Tsuboi A, Oka Y, Nakajima H, Fukuda Y, Elisseeva OA, Yoshihara S (2007). Wilms tumor gene WT1 peptide-based immunotherapy induced a minimal response in a patient with advanced therapy-resistant multiple myeloma. Int. J. Hematol.

[b45] Keilholz U, Letsch A, Busse A, Asemissen AM, Bauer S, Blau IW (2009). A clinical and immunologic phase 2 trial of Wilms tumor gene product 1 (WT1) peptide vaccination in patients with AML and MDS. Blood.

[b46] Tsuboi A, Oka Y, Osaki T, Kumagai T, Tachibana I, Hayashi S (2004). WT1 peptide-based immunotherapy for patients with lung cancer: report of two cases. Microbiol. Immunol.

[b47] Krug LM, Dao T, Brown AB, Maslak P, Travis W, Bekele S (2010). WT1 peptide vaccinations induce CD4 and CD8 T cell immune responses in patients with mesothelioma and non-small cell lung cancer. Cancer Immunol. Immunother.

[b48] Dao T, Yan S, Veomett N, Pankov D, Zhou L, Korontsvit T (2013). Targeting the intracellular WT1 oncogene product with a therapeutic human antibody. Sci. Transl. Med.

[b49] Netinatsunthorn W, Hanprasertpong J, Dechsukhum C, Leetanaporn R, Geater A (2006). WT1 gene expression as a prognostic marker in advanced serous epithelial ovarian carcinoma: an immunohistochemical study. BMC Cancer.

[b50] Hwang H, Quenneville L, Yaziji H, Gown AM (2004). Wilms tumor gene product: sensitive and contextually specific marker of serous carcinomas of ovarian surface epithelial origin. Appl. Immunohistochem. Mol. Morphol.

[b51] Waldstrom M, Grove A (2005). Immunohistochemical expression of Wilms tumor gene protein in different histologic subtypes of ovarian carcinomas. Arch. Pathol. Lab. Med.

